# Supplementary material for: Effectiveness and Safety of Ayurvedic Medicines in Type 2 Diabetes Mellitus Management: A Systematic Review and Meta-Analysis
Source: Front Pharmacol. 2022 Jun 8;13:821810. doi: 10.3389/fphar.2022.821810 (PMC9213670; doi:10.3389/fphar.2022.821810)
Supplement: Supplementary file 1 [file DataSheet1.zip › Supplementary Material/Forest and Funnel Plots/Gynostemma pentaphyllum/Stimulated insulin.pdf]

## Gynostemma pentaphyllum - Stimulated insulin

| | Intervention | | | Comparator | | | Mean Diff. | Weight |
|---|---|---|---|---|---|---|---|---|
| Study | N | Mean | SD | N | Mean | SD | with 95% CI | (%) |
| Huyen 2010 | 11 | 14.14 | 18.66 | 12 | 26.71 | 20.16 | -12.57 [ -28.49, 3.35] | 59.15 |
| Huyen 2012 | 12 | 1.04 | 38.97 | 13 | -9.85 | 24.54 | 10.89 [ -14.42, 36.20] | 40.85 |
| **Overall** | | | | | | | -2.99 [ -25.59, 19.61] | |

Heterogeneity: $\tau^2 = 158.82$, $I^2 = 57.71\%$, $H^2 = 2.36$

Test of $\theta_i = \theta_j$: Q(1) = 2.36, p = 0.12

Test of $\theta = 0$: z = -0.26, p = 0.80

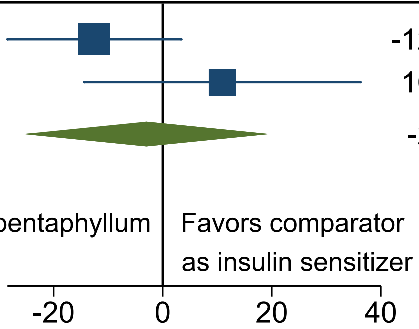


Random-effects REML model
